# Diaqua­bis(1,10-phenanthroline-κ^2^ *N*,*N*′)zinc(II) 2-hydr­oxy-5-sulfonatobenzoate tetra­hydrate

**DOI:** 10.1107/S1600536809003055

**Published:** 2009-01-31

**Authors:** Kou-Lin Zhang, Bo Yang, Seik Weng Ng

**Affiliations:** a College of Chemistry and Chemical Engineering, Yangzhou University, Yangzhou 225002, People’s Republic of China; b Department of Chemistry, University of Malaya, 50603 Kuala Lumpur, Malaysia

## Abstract

The water-coordinated metal centre in the title salt, [Zn(C_12_H_8_N_2_)_2_(H_2_O)_2_]C_7_H_4_O_6_S·4H_2_O, is chelated by the two bidentate *N*-heterocycles, leading to an overall distorted octa­hedral environment. The cation, dianion and solvent water mol­ecules inter­act by O—H⋯O hydrogen bonds to form a layer motif. The SO_3_ group is disordered over two positions with respect to the O atoms in a 0.76 (1):0.24 (1) ratio. One of the solvent water molecules is also disordered over two positions in a 0.56 (4):0.44 (4) ratio.

## Related literature

For the isostructural manganese(II), nickel(II) and cobalt(II) analogues, see: Fan *et al.* (2005 ▶); Chen *et al.* (2005 ▶); Zhu & Fan (2005 ▶).
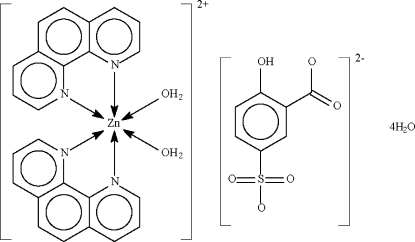


## Experimental

### 

#### Crystal data

[Zn(C_12_H_8_N_2_)_2_(H_2_O)_2_]C_7_H_4_O_6_S·4H_2_O
*M* *_r_* = 750.04
Triclinic, 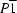

*a* = 10.075 (1) Å
*b* = 12.263 (1) Å
*c* = 13.927 (1) Å
α = 96.937 (2)°
β = 101.495 (2)°
γ = 98.856 (2)°
*V* = 1645.5 (2) Å^3^
*Z* = 2
Mo *K*α radiation
μ = 0.88 mm^−1^
*T* = 293 (2) K
0.38 × 0.30 × 0.22 mm

#### Data collection

Bruker APEXII area-detector diffractometer
Absorption correction: multi-scan (*SADABS*; Sheldrick, 1996 ▶) *T*_min_ = 0.36, *T*_max_ = 0.82
10806 measured reflections
7315 independent reflections
4849 reflections with *I* > 2σ(*I*)
*R*_int_ = 0.020

#### Refinement

*R*[*F* ^2^ > 2σ(*F* ^2^)] = 0.037
*wR*(*F* ^2^) = 0.095
*S* = 0.93
7315 reflections
513 parameters
106 restraints
H atoms treated by a mixture of independent and constrained refinement
Δρ_max_ = 0.28 e Å^−3^
Δρ_min_ = −0.35 e Å^−3^

### 

Data collection: *APEX2* (Bruker, 2004 ▶); cell refinement: *SAINT* (Bruker, 2004 ▶); data reduction: *SAINT*; program(s) used to solve structure: *SHELXS97* (Sheldrick, 2008 ▶); program(s) used to refine structure: *SHELXL97* (Sheldrick, 2008 ▶); molecular graphics: *X-SEED* (Barbour, 2001 ▶); software used to prepare material for publication: *publCIF* (Westrip, 2009 ▶).

## Supplementary Material

Crystal structure: contains datablocks global, I. DOI: 10.1107/S1600536809003055/xu2475sup1.cif

Structure factors: contains datablocks I. DOI: 10.1107/S1600536809003055/xu2475Isup2.hkl

Additional supplementary materials:  crystallographic information; 3D view; checkCIF report

## Figures and Tables

**Table 1:** Hydrogen-bond geometry (Å, °)

*D*—H⋯*A*	*D*—H	H⋯*A*	*D*⋯*A*	*D*—H⋯*A*
O1*W*—H11⋯O1	0.83 (1)	1.81 (1)	2.632 (2)	170 (3)
O1*W*—H12⋯O6^i^	0.84 (1)	1.95 (1)	2.793 (4)	177 (2)
O1*W*—H12⋯O6′^i^	0.84 (1)	2.04 (2)	2.787 (7)	147 (2)
O2*W*—H21⋯O3*W*	0.85 (1)	1.87 (1)	2.714 (3)	170 (3)
O2*W*—H22⋯O6*W*	0.85 (1)	1.95 (1)	2.778 (6)	166 (3)
O2*W*—H22⋯O6*W*′	0.85 (1)	1.80 (2)	2.615 (6)	160 (3)
O3*W*—H31⋯O5*W*	0.85 (1)	1.91 (1)	2.754 (3)	173 (3)
O3*W*—H32⋯O5^ii^	0.85 (1)	1.95 (1)	2.805 (4)	178 (3)
O3*W*—H32⋯O5′^ii^	0.85 (1)	2.08 (2)	2.892 (10)	159 (3)
O4*W*—H41⋯O4^iii^	0.86 (1)	2.17 (3)	2.962 (5)	153 (5)
O4*W*—H41⋯O4′^iii^	0.86 (1)	1.74 (2)	2.598 (8)	173 (5)
O4*W*—H42⋯O5^ii^	0.86 (1)	2.22 (1)	3.065 (5)	169 (5)
O4*W*—H42⋯O5′^ii^	0.86 (1)	2.08 (2)	2.869 (15)	153 (4)
O5*W*—H51⋯O4^iii^	0.84 (1)	2.07 (1)	2.900 (4)	168 (4)
O5*W*—H51⋯O6′^iii^	0.84 (1)	2.07 (2)	2.823 (8)	148 (3)
O5*W*—H52⋯O2^iv^	0.85 (1)	1.94 (1)	2.792 (3)	175 (3)
O6*W*—H61⋯O6^i^	0.83 (1)	2.22 (3)	2.751 (8)	122 (3)
O6*W*—H62⋯O4*W*	0.84 (1)	1.88 (2)	2.642 (7)	150 (4)

Symmetry codes: (i) 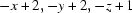; (ii) 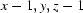; (iii) 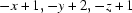; (iv) 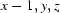.

**Fig. 1.**
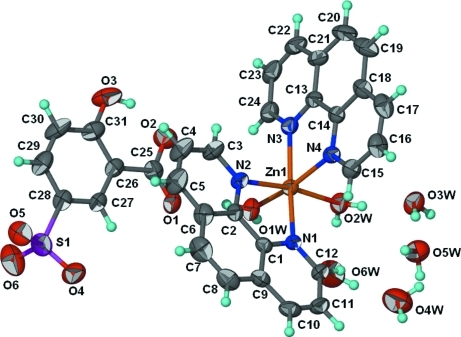
Thermal ellipsoid plot of [Zn(OH2)2(C12H8N2)2][C7H4O6S].4H2O; displacement ellipsoids are drawn at the 50% probability level, and H atoms as spheres of arbitrary radii. The disorder is not shown.

## supplementary crystallographic information

### Experimental

1,10-Phenanthroline monohydrate (0.10 g, 0.5 mmol) was dissolved in methanol (10 ml). To this solution was added zinc nitrate hexahydrate (0.15 g, 0.5 mmol) dissolved in water (10 ml). The solution was then mixed with an aqueous solution of 5-sulfosalicylic acid (0.11 g, 0.5 mmol) and sodium hydroxide (0.04 g, 1 mmol). Crystals separated after several days. These were collected and washed with methanol; yield: 50%. CH&N elemental analysis: Calc. for C_31_H_32_N_4_O_12_SZn: C 49.63, H 4.27, N 7.47%. Found: C 49.71, H 4.31, N, 7.41%.

### Refinement

The –SO_3_ group is disordered over two positions with respect to the O atoms. The S—O distances were restrained to 0.01 Å of each other, as were the O···O distances. The anisotropic temperature factors were restrained to be nearly isotropic. The disordered refined to a 0.76 (1):0.24 ratio. One of the lattice water molecules is also disordered over two positions in a 0.56 (4):0.44 ratio. The temperature factors of the two components were restrained to be equal to each other. The anisotropic temperature factors were also restrained to be nearly isotropic.

The carbon-bound H atoms were placed in calculated positions and were allowed to ride on the parent atoms. The oxygen-bound ones were located in a difference Fourier map, and were refined with distance restraints O—H = 0.85 (1) and H···H = 1.39 (1) Å. Their temperature factors were tied by a factor of 1.5.

### Crystal data

**Table:**

[Zn(C_12_H_8_N_2_)_2_(H_2_O)_2_]C_7_H_4_O_6_S·4H_2_O	*Z* = 2
*M**_r_* = 750.04	*F*(000) = 776
Triclinic, *P*1	*D*_x_ = 1.514 Mg m^−^^3^
Hall symbol: -P 1	Mo *K*α radiation, λ = 0.71073 Å
*a* = 10.075 (1) Å	Cell parameters from 2300 reflections
*b* = 12.263 (1) Å	θ = 2.5–22.9°
*c* = 13.927 (1) Å	µ = 0.88 mm^−^^1^
α = 96.937 (2)°	*T* = 293 K
β = 101.495 (2)°	Block, colourless
γ = 98.856 (2)°	0.38 × 0.30 × 0.22 mm
*V* = 1645.5 (2) Å^3^	

### Data collection

**Table:**

Bruker APEXII area-detector diffractometer	7315 independent reflections
Radiation source: fine-focus sealed tube	4849 reflections with *I* > 2σ(*I*)
graphite	*R*_int_ = 0.020
φ and ω scans	θ_max_ = 27.5°, θ_min_ = 1.7°
Absorption correction: multi-scan (*SADABS*; Sheldrick, 1996)	*h* = −13→12
*T*_min_ = 0.36, *T*_max_ = 0.82	*k* = −14→15
10806 measured reflections	*l* = −18→15

### Refinement

**Table:**

Refinement on *F*^2^	Primary atom site location: structure-invariant direct methods
Least-squares matrix: full	Secondary atom site location: difference Fourier map
*R*[*F*^2^ > 2σ(*F*^2^)] = 0.037	Hydrogen site location: inferred from neighbouring sites
*wR*(*F*^2^) = 0.095	H atoms treated by a mixture of independent and constrained refinement
*S* = 0.93	*w* = 1/[σ^2^(*F*_o_^2^) + (0.0512*P*)^2^] where *P* = (*F*_o_^2^ + 2*F*_c_^2^)/3
7315 reflections	(Δ/σ)_max_ = 0.001
513 parameters	Δρ_max_ = 0.28 e Å^−^^3^
106 restraints	Δρ_min_ = −0.35 e Å^−^^3^

### Fractional atomic coordinates and isotropic or equivalent isotropic displacement parameters (Å^2^)

**Table:**

	*x*	*y*	*z*	*U*_iso_*/*U*_eq_	Occ. (<1)
Zn1	0.50705 (3)	0.73388 (2)	0.26312 (2)	0.03952 (10)	
S1	1.09039 (9)	0.91846 (6)	0.82131 (5)	0.0656 (2)	
N1	0.40757 (18)	0.83635 (15)	0.35340 (14)	0.0376 (4)	
N2	0.5761 (2)	0.69351 (16)	0.40972 (14)	0.0417 (5)	
N3	0.5781 (2)	0.59926 (17)	0.18305 (14)	0.0439 (5)	
N4	0.32841 (19)	0.59452 (15)	0.22700 (14)	0.0402 (5)	
O1	0.9002 (2)	0.85085 (16)	0.42946 (13)	0.0634 (5)	
O2	0.9822 (2)	0.70605 (17)	0.36560 (14)	0.0701 (6)	
O3	1.1172 (3)	0.59708 (19)	0.47739 (18)	0.0844 (7)	
H3	1.064 (4)	0.613 (4)	0.428 (2)	0.127*	
O4	0.9776 (4)	0.9750 (3)	0.7937 (2)	0.0854 (15)	0.757 (7)
O5	1.0879 (5)	0.8618 (3)	0.9044 (3)	0.0724 (11)	0.757 (7)
O6	1.2238 (5)	0.9995 (3)	0.8373 (3)	0.1028 (16)	0.757 (7)
O4'	0.9391 (7)	0.8922 (10)	0.8218 (8)	0.104 (5)	0.243 (7)
O5'	1.1646 (14)	0.8780 (11)	0.9059 (9)	0.101 (5)	0.243 (7)
O6'	1.1326 (10)	1.0279 (5)	0.8124 (7)	0.054 (3)	0.243 (7)
O1W	0.69078 (17)	0.84787 (16)	0.28148 (14)	0.0517 (4)	
H11	0.757 (2)	0.841 (2)	0.3255 (15)	0.078*	
H12	0.713 (3)	0.8936 (19)	0.2443 (16)	0.078*	
O2W	0.42757 (19)	0.79208 (16)	0.13328 (14)	0.0555 (5)	
H21	0.3404 (11)	0.779 (2)	0.114 (2)	0.083*	
H22	0.459 (2)	0.8582 (13)	0.127 (2)	0.083*	
O3W	0.1536 (2)	0.73101 (18)	0.05280 (15)	0.0658 (5)	
H31	0.103 (3)	0.742 (3)	0.0938 (18)	0.099*	
H32	0.134 (3)	0.770 (2)	0.0068 (16)	0.099*	
O4W	0.2383 (3)	1.0300 (3)	0.0895 (2)	0.1163 (9)	
H41	0.182 (4)	1.052 (4)	0.123 (3)	0.174*	
H42	0.190 (4)	0.990 (4)	0.035 (2)	0.174*	
O5W	−0.0022 (2)	0.78419 (18)	0.18724 (16)	0.0698 (6)	
H51	−0.008 (4)	0.8523 (12)	0.190 (2)	0.105*	
H52	−0.010 (4)	0.763 (2)	0.2422 (14)	0.105*	
O6W	0.5032 (7)	1.0198 (5)	0.1338 (4)	0.0941 (15)	0.54 (6)
O6W'	0.4960 (7)	0.9808 (5)	0.0703 (5)	0.0941 (15)	0.46
H61	0.5655 (17)	1.030 (3)	0.1029 (18)	0.141*	
H62	0.429 (2)	1.038 (4)	0.105 (3)	0.141*	
C1	0.4359 (2)	0.82539 (18)	0.45066 (17)	0.0364 (5)	
C2	0.5265 (2)	0.74973 (19)	0.48134 (17)	0.0387 (5)	
C3	0.6591 (3)	0.6232 (2)	0.4370 (2)	0.0579 (7)	
H3A	0.6932	0.5842	0.3887	0.069*	
C4	0.6975 (3)	0.6053 (3)	0.5344 (3)	0.0764 (9)	
H4	0.7556	0.5550	0.5505	0.092*	
C5	0.6491 (3)	0.6622 (3)	0.6058 (2)	0.0751 (9)	
H5	0.6747	0.6512	0.6713	0.090*	
C6	0.5605 (3)	0.7376 (2)	0.58146 (19)	0.0545 (7)	
C7	0.5041 (3)	0.8011 (3)	0.6521 (2)	0.0681 (8)	
H7	0.5275	0.7939	0.7188	0.082*	
C8	0.4186 (3)	0.8704 (2)	0.6232 (2)	0.0589 (8)	
H8	0.3832	0.9101	0.6703	0.071*	
C9	0.3807 (2)	0.88451 (19)	0.52230 (19)	0.0443 (6)	
C10	0.2908 (3)	0.9557 (2)	0.4877 (2)	0.0531 (7)	
H10	0.2512	0.9960	0.5317	0.064*	
C11	0.2621 (3)	0.9652 (2)	0.3902 (2)	0.0542 (7)	
H11A	0.2022	1.0116	0.3669	0.065*	
C12	0.3231 (2)	0.90484 (19)	0.3246 (2)	0.0461 (6)	
H12A	0.3035	0.9132	0.2581	0.055*	
C13	0.4829 (3)	0.50289 (19)	0.15053 (16)	0.0411 (6)	
C14	0.3499 (2)	0.50073 (19)	0.17326 (16)	0.0407 (5)	
C15	0.2076 (3)	0.5927 (2)	0.25028 (19)	0.0496 (6)	
H15	0.1927	0.6562	0.2873	0.060*	
C16	0.1008 (3)	0.4998 (2)	0.2220 (2)	0.0602 (7)	
H16	0.0175	0.5020	0.2407	0.072*	
C17	0.1194 (3)	0.4070 (2)	0.1673 (2)	0.0624 (8)	
H17	0.0484	0.3453	0.1472	0.075*	
C18	0.2475 (3)	0.4041 (2)	0.14070 (18)	0.0516 (7)	
C19	0.2778 (4)	0.3086 (2)	0.0854 (2)	0.0712 (9)	
H19	0.2102	0.2448	0.0633	0.085*	
C20	0.4028 (4)	0.3099 (2)	0.0649 (2)	0.0696 (9)	
H20	0.4206	0.2463	0.0299	0.084*	
C21	0.5102 (3)	0.4076 (2)	0.09595 (18)	0.0533 (7)	
C22	0.6413 (3)	0.4162 (3)	0.0741 (2)	0.0641 (8)	
H22A	0.6641	0.3555	0.0382	0.077*	
C23	0.7349 (3)	0.5124 (3)	0.1051 (2)	0.0644 (8)	
H23	0.8214	0.5180	0.0901	0.077*	
C24	0.7002 (3)	0.6032 (2)	0.15969 (19)	0.0551 (7)	
H24	0.7652	0.6687	0.1805	0.066*	
C25	0.9671 (3)	0.7750 (2)	0.4384 (2)	0.0512 (6)	
C26	1.0362 (2)	0.7593 (2)	0.54018 (19)	0.0449 (6)	
C27	1.0324 (2)	0.8337 (2)	0.62312 (18)	0.0442 (6)	
H27	0.9882	0.8942	0.6145	0.053*	
C28	1.0928 (3)	0.8198 (2)	0.71780 (19)	0.0484 (6)	
C29	1.1577 (3)	0.7286 (3)	0.7311 (2)	0.0644 (8)	
H29	1.1969	0.7177	0.7947	0.077*	
C30	1.1638 (3)	0.6547 (3)	0.6508 (3)	0.0714 (9)	
H30	1.2075	0.5941	0.6603	0.086*	
C31	1.1054 (3)	0.6693 (2)	0.5552 (2)	0.0573 (7)	

### Atomic displacement parameters (Å^2^)

**Table:**

	*U*^11^	*U*^22^	*U*^33^	*U*^12^	*U*^13^	*U*^23^
Zn1	0.04032 (17)	0.04133 (17)	0.03760 (16)	0.01290 (12)	0.00851 (12)	0.00213 (11)
S1	0.0877 (6)	0.0620 (5)	0.0438 (4)	0.0094 (4)	0.0046 (4)	0.0170 (4)
N1	0.0359 (11)	0.0355 (10)	0.0403 (11)	0.0103 (8)	0.0060 (9)	0.0005 (9)
N2	0.0415 (11)	0.0416 (11)	0.0453 (12)	0.0148 (9)	0.0107 (9)	0.0085 (9)
N3	0.0423 (12)	0.0524 (12)	0.0394 (11)	0.0176 (10)	0.0097 (9)	0.0041 (9)
N4	0.0406 (11)	0.0406 (11)	0.0409 (11)	0.0097 (9)	0.0107 (9)	0.0062 (9)
O1	0.0622 (12)	0.0758 (13)	0.0498 (11)	0.0292 (11)	−0.0026 (9)	0.0050 (10)
O2	0.0863 (15)	0.0732 (13)	0.0538 (12)	0.0241 (11)	0.0205 (11)	0.0014 (10)
O3	0.116 (2)	0.0723 (14)	0.0890 (18)	0.0524 (14)	0.0495 (16)	0.0187 (13)
O4	0.123 (3)	0.084 (3)	0.0535 (18)	0.055 (2)	0.0025 (17)	0.0085 (16)
O5	0.106 (3)	0.068 (2)	0.0442 (17)	0.009 (2)	0.0146 (19)	0.0256 (15)
O6	0.113 (3)	0.083 (2)	0.088 (2)	−0.029 (2)	−0.010 (2)	0.0347 (19)
O4'	0.100 (7)	0.099 (8)	0.115 (8)	−0.011 (5)	0.075 (6)	−0.024 (6)
O5'	0.139 (10)	0.096 (8)	0.065 (7)	0.025 (7)	0.007 (7)	0.026 (5)
O6'	0.066 (6)	0.046 (4)	0.064 (5)	0.021 (4)	0.025 (4)	0.025 (4)
O1W	0.0425 (10)	0.0623 (12)	0.0495 (11)	0.0061 (9)	0.0048 (8)	0.0192 (9)
O2W	0.0574 (11)	0.0635 (12)	0.0460 (11)	0.0180 (10)	0.0058 (10)	0.0111 (10)
O3W	0.0660 (13)	0.0783 (14)	0.0580 (13)	0.0261 (11)	0.0105 (10)	0.0185 (11)
O4W	0.145 (3)	0.096 (2)	0.099 (2)	0.0271 (19)	0.0184 (19)	−0.0103 (16)
O5W	0.0734 (14)	0.0677 (13)	0.0767 (15)	0.0189 (12)	0.0283 (12)	0.0169 (11)
O6W	0.115 (3)	0.091 (3)	0.078 (3)	0.013 (2)	0.024 (3)	0.023 (3)
O6W'	0.115 (3)	0.091 (3)	0.078 (3)	0.013 (2)	0.024 (3)	0.023 (3)
C1	0.0338 (12)	0.0333 (12)	0.0398 (13)	0.0011 (10)	0.0098 (10)	0.0001 (10)
C2	0.0371 (13)	0.0393 (13)	0.0390 (13)	0.0047 (10)	0.0080 (10)	0.0070 (10)
C3	0.0621 (18)	0.0594 (17)	0.0632 (19)	0.0307 (15)	0.0179 (15)	0.0210 (14)
C4	0.084 (2)	0.083 (2)	0.080 (2)	0.0452 (19)	0.0197 (19)	0.0421 (19)
C5	0.087 (2)	0.094 (2)	0.0559 (19)	0.032 (2)	0.0137 (17)	0.0404 (18)
C6	0.0597 (17)	0.0634 (17)	0.0434 (15)	0.0095 (14)	0.0134 (13)	0.0190 (13)
C7	0.084 (2)	0.084 (2)	0.0392 (16)	0.0082 (18)	0.0221 (15)	0.0144 (15)
C8	0.0627 (19)	0.0658 (18)	0.0488 (17)	0.0029 (15)	0.0277 (15)	−0.0032 (14)
C9	0.0404 (14)	0.0418 (13)	0.0487 (15)	0.0005 (11)	0.0170 (12)	−0.0036 (11)
C10	0.0445 (15)	0.0436 (14)	0.072 (2)	0.0066 (12)	0.0261 (14)	−0.0097 (13)
C11	0.0415 (15)	0.0450 (15)	0.076 (2)	0.0176 (12)	0.0113 (14)	0.0008 (14)
C12	0.0420 (14)	0.0425 (14)	0.0535 (16)	0.0158 (11)	0.0050 (12)	0.0060 (12)
C13	0.0552 (15)	0.0397 (13)	0.0308 (12)	0.0174 (12)	0.0069 (11)	0.0088 (10)
C14	0.0516 (15)	0.0383 (13)	0.0314 (12)	0.0094 (11)	0.0043 (11)	0.0095 (10)
C15	0.0439 (15)	0.0537 (16)	0.0527 (16)	0.0095 (12)	0.0134 (12)	0.0091 (12)
C16	0.0488 (17)	0.0710 (19)	0.0586 (18)	0.0008 (14)	0.0124 (14)	0.0139 (15)
C17	0.0621 (19)	0.0613 (18)	0.0537 (18)	−0.0128 (15)	0.0043 (14)	0.0145 (15)
C18	0.0693 (19)	0.0425 (14)	0.0380 (14)	0.0025 (13)	0.0045 (13)	0.0095 (11)
C19	0.106 (3)	0.0404 (16)	0.059 (2)	0.0000 (16)	0.0144 (19)	0.0007 (14)
C20	0.114 (3)	0.0418 (16)	0.0536 (18)	0.0264 (17)	0.0167 (19)	−0.0018 (13)
C21	0.083 (2)	0.0474 (15)	0.0351 (14)	0.0336 (15)	0.0106 (13)	0.0069 (12)
C22	0.092 (2)	0.073 (2)	0.0411 (16)	0.0530 (19)	0.0180 (16)	0.0075 (14)
C23	0.0593 (18)	0.101 (2)	0.0461 (17)	0.0459 (18)	0.0169 (14)	0.0138 (16)
C24	0.0457 (15)	0.0748 (19)	0.0482 (16)	0.0234 (14)	0.0118 (13)	0.0053 (14)
C25	0.0460 (15)	0.0575 (16)	0.0501 (16)	0.0075 (13)	0.0133 (13)	0.0064 (13)
C26	0.0374 (13)	0.0493 (14)	0.0539 (16)	0.0117 (11)	0.0170 (12)	0.0151 (12)
C27	0.0372 (13)	0.0491 (14)	0.0500 (15)	0.0132 (11)	0.0106 (11)	0.0139 (12)
C28	0.0452 (15)	0.0537 (15)	0.0515 (16)	0.0091 (12)	0.0146 (12)	0.0216 (13)
C29	0.0629 (19)	0.078 (2)	0.066 (2)	0.0272 (16)	0.0183 (15)	0.0413 (17)
C30	0.082 (2)	0.071 (2)	0.088 (2)	0.0460 (18)	0.0382 (19)	0.0433 (19)
C31	0.0589 (17)	0.0553 (16)	0.070 (2)	0.0202 (14)	0.0297 (15)	0.0212 (15)

### Geometric parameters (Å, °)

**Table:**

Zn1—O2W	2.0810 (18)	C4—C5	1.358 (4)
Zn1—O1W	2.0914 (17)	C4—H4	0.9300
Zn1—N1	2.1615 (17)	C5—C6	1.406 (4)
Zn1—N2	2.164 (2)	C5—H5	0.9300
Zn1—N3	2.1720 (18)	C6—C7	1.438 (4)
Zn1—N4	2.2130 (19)	C7—C8	1.341 (4)
S1—O6'	1.372 (5)	C7—H7	0.9300
S1—O5	1.422 (3)	C8—C9	1.420 (4)
S1—O4	1.433 (3)	C8—H8	0.9300
S1—O5'	1.448 (6)	C9—C10	1.409 (4)
S1—O6	1.503 (3)	C10—C11	1.354 (4)
S1—O4'	1.511 (6)	C10—H10	0.9300
S1—C28	1.773 (3)	C11—C12	1.400 (3)
N1—C12	1.325 (3)	C11—H11A	0.9300
N1—C1	1.355 (3)	C12—H12A	0.9300
N2—C3	1.327 (3)	C13—C21	1.410 (3)
N2—C2	1.363 (3)	C13—C14	1.434 (3)
N3—C24	1.330 (3)	C14—C18	1.407 (3)
N3—C13	1.367 (3)	C15—C16	1.398 (4)
N4—C15	1.318 (3)	C15—H15	0.9300
N4—C14	1.365 (3)	C16—C17	1.351 (4)
O1—C25	1.234 (3)	C16—H16	0.9300
O2—C25	1.288 (3)	C17—C18	1.417 (4)
O3—C31	1.351 (3)	C17—H17	0.9300
O3—H3	0.853 (11)	C18—C19	1.430 (4)
O1W—H11	0.832 (10)	C19—C20	1.344 (4)
O1W—H12	0.843 (10)	C19—H19	0.9300
O2W—H21	0.849 (10)	C20—C21	1.443 (4)
O2W—H22	0.845 (10)	C20—H20	0.9300
O3W—H31	0.849 (10)	C21—C22	1.405 (4)
O3W—H32	0.853 (10)	C22—C23	1.356 (4)
O4W—H41	0.859 (11)	C22—H22A	0.9300
O4W—H42	0.858 (11)	C23—C24	1.400 (4)
O5W—H51	0.844 (10)	C23—H23	0.9300
O5W—H52	0.851 (10)	C24—H24	0.9300
O6W—O6W'	0.937 (6)	C25—C26	1.498 (4)
O6W—H61	0.831 (11)	C26—C27	1.394 (3)
O6W—H62	0.843 (8)	C26—C31	1.408 (3)
O6W'—H61	0.857 (11)	C27—C28	1.380 (3)
O6W'—H62	1.17 (3)	C27—H27	0.9300
C1—C9	1.415 (3)	C28—C29	1.393 (4)
C1—C2	1.441 (3)	C29—C30	1.369 (4)
C2—C6	1.400 (3)	C29—H29	0.9300
C3—C4	1.388 (4)	C30—C31	1.388 (4)
C3—H3A	0.9300	C30—H30	0.9300
			
O2W—Zn1—O1W	89.83 (7)	C2—C6—C5	116.4 (2)
O2W—Zn1—N1	93.87 (7)	C2—C6—C7	119.3 (3)
O1W—Zn1—N1	97.42 (7)	C5—C6—C7	124.3 (3)
O2W—Zn1—N2	171.00 (7)	C8—C7—C6	121.0 (3)
O1W—Zn1—N2	90.11 (7)	C8—C7—H7	119.5
N1—Zn1—N2	77.21 (7)	C6—C7—H7	119.5
O2W—Zn1—N3	92.55 (7)	C7—C8—C9	121.5 (2)
O1W—Zn1—N3	94.26 (8)	C7—C8—H8	119.2
N1—Zn1—N3	166.67 (7)	C9—C8—H8	119.2
N2—Zn1—N3	96.43 (7)	C10—C9—C1	116.7 (2)
O2W—Zn1—N4	89.26 (7)	C10—C9—C8	124.0 (2)
O1W—Zn1—N4	170.12 (7)	C1—C9—C8	119.4 (2)
N1—Zn1—N4	92.46 (7)	C11—C10—C9	119.9 (2)
N2—Zn1—N4	92.32 (7)	C11—C10—H10	120.0
N3—Zn1—N4	75.96 (7)	C9—C10—H10	120.0
O6'—S1—O5	133.1 (4)	C10—C11—C12	119.6 (2)
O6'—S1—O4	67.2 (4)	C10—C11—H11A	120.2
O5—S1—O4	115.8 (2)	C12—C11—H11A	120.2
O6'—S1—O5'	116.8 (5)	N1—C12—C11	122.8 (2)
O5—S1—O5'	30.8 (5)	N1—C12—H12A	118.6
O4—S1—O5'	140.9 (7)	C11—C12—H12A	118.6
O6'—S1—O6	42.2 (3)	N3—C13—C21	122.9 (2)
O5—S1—O6	110.5 (2)	N3—C13—C14	117.6 (2)
O4—S1—O6	109.3 (2)	C21—C13—C14	119.5 (2)
O5'—S1—O6	82.5 (5)	N4—C14—C18	122.4 (2)
O6'—S1—O4'	112.4 (5)	N4—C14—C13	117.5 (2)
O5—S1—O4'	76.4 (5)	C18—C14—C13	120.0 (2)
O4—S1—O4'	46.8 (5)	N4—C15—C16	123.2 (2)
O5'—S1—O4'	107.2 (5)	N4—C15—H15	118.4
O6—S1—O4'	151.4 (4)	C16—C15—H15	118.4
O6'—S1—C28	114.8 (3)	C17—C16—C15	119.5 (3)
O5—S1—C28	108.38 (19)	C17—C16—H16	120.2
O4—S1—C28	108.01 (15)	C15—C16—H16	120.2
O5'—S1—C28	104.5 (6)	C16—C17—C18	119.7 (3)
O6—S1—C28	104.2 (2)	C16—C17—H17	120.2
O4'—S1—C28	99.3 (4)	C18—C17—H17	120.2
C12—N1—C1	118.06 (19)	C14—C18—C17	117.0 (2)
C12—N1—Zn1	128.06 (16)	C14—C18—C19	119.4 (3)
C1—N1—Zn1	113.88 (14)	C17—C18—C19	123.6 (3)
C3—N2—C2	117.8 (2)	C20—C19—C18	120.8 (3)
C3—N2—Zn1	128.40 (17)	C20—C19—H19	119.6
C2—N2—Zn1	113.79 (15)	C18—C19—H19	119.6
C24—N3—C13	118.0 (2)	C19—C20—C21	121.7 (3)
C24—N3—Zn1	126.88 (18)	C19—C20—H20	119.2
C13—N3—Zn1	115.07 (15)	C21—C20—H20	119.2
C15—N4—C14	118.1 (2)	C22—C21—C13	116.6 (3)
C15—N4—Zn1	128.09 (16)	C22—C21—C20	124.7 (2)
C14—N4—Zn1	113.80 (15)	C13—C21—C20	118.6 (3)
C31—O3—H3	104 (3)	C23—C22—C21	120.4 (2)
Zn1—O1W—H11	117.2 (17)	C23—C22—H22A	119.8
Zn1—O1W—H12	129.0 (17)	C21—C22—H22A	119.8
H11—O1W—H12	112.9 (17)	C22—C23—C24	119.5 (3)
Zn1—O2W—H21	116 (2)	C22—C23—H23	120.3
Zn1—O2W—H22	118 (2)	C24—C23—H23	120.3
H21—O2W—H22	109.6 (16)	N3—C24—C23	122.6 (3)
H31—O3W—H32	108.2 (16)	N3—C24—H24	118.7
H41—O4W—H42	107.2 (18)	C23—C24—H24	118.7
H51—O5W—H52	109.5 (17)	O1—C25—O2	124.6 (3)
O6W'—O6W—H61	57.6 (9)	O1—C25—C26	119.1 (2)
O6W'—O6W—H62	82 (3)	O2—C25—C26	116.2 (2)
H61—O6W—H62	113.0 (18)	C27—C26—C31	118.1 (2)
O6W—O6W'—H61	54.9 (9)	C27—C26—C25	120.4 (2)
O6W—O6W'—H62	45.5 (13)	C31—C26—C25	121.5 (2)
H61—O6W'—H62	86 (2)	C28—C27—C26	121.5 (2)
N1—C1—C9	123.0 (2)	C28—C27—H27	119.2
N1—C1—C2	117.85 (19)	C26—C27—H27	119.2
C9—C1—C2	119.2 (2)	C27—C28—C29	119.3 (3)
N2—C2—C6	123.1 (2)	C27—C28—S1	120.31 (19)
N2—C2—C1	117.3 (2)	C29—C28—S1	120.4 (2)
C6—C2—C1	119.6 (2)	C30—C29—C28	120.3 (3)
N2—C3—C4	123.0 (3)	C30—C29—H29	119.9
N2—C3—H3A	118.5	C28—C29—H29	119.9
C4—C3—H3A	118.5	C29—C30—C31	120.7 (3)
C5—C4—C3	119.0 (3)	C29—C30—H30	119.6
C5—C4—H4	120.5	C31—C30—H30	119.6
C3—C4—H4	120.5	O3—C31—C30	119.2 (3)
C4—C5—C6	120.6 (3)	O3—C31—C26	120.8 (3)
C4—C5—H5	119.7	C30—C31—C26	120.0 (3)
C6—C5—H5	119.7		

### Hydrogen-bond geometry (Å, °)

**Table:**

*D*—H···*A*	*D*—H	H···*A*	*D*···*A*	*D*—H···*A*
O1W—H11···O1	0.83 (1)	1.81 (1)	2.632 (2)	170 (3)
O1W—H12···O6^i^	0.84 (1)	1.95 (1)	2.793 (4)	177 (2)
O1W—H12···O6'^i^	0.84 (1)	2.04 (2)	2.787 (7)	147 (2)
O2W—H21···O3W	0.85 (1)	1.87 (1)	2.714 (3)	170 (3)
O2W—H22···O6W	0.85 (1)	1.95 (1)	2.778 (6)	166 (3)
O2W—H22···O6W'	0.85 (1)	1.80 (2)	2.615 (6)	160 (3)
O3W—H31···O5W	0.85 (1)	1.91 (1)	2.754 (3)	173 (3)
O3W—H32···O5^ii^	0.85 (1)	1.95 (1)	2.805 (4)	178 (3)
O3W—H32···O5'^ii^	0.85 (1)	2.08 (2)	2.892 (10)	159 (3)
O4W—H41···O4^iii^	0.86 (1)	2.17 (3)	2.962 (5)	153 (5)
O4W—H41···O4'^iii^	0.86 (1)	1.74 (2)	2.598 (8)	173 (5)
O4W—H42···O5^ii^	0.86 (1)	2.22 (1)	3.065 (5)	169 (5)
O4W—H42···O5'^ii^	0.86 (1)	2.08 (2)	2.869 (15)	153 (4)
O5W—H51···O4^iii^	0.84 (1)	2.07 (1)	2.900 (4)	168 (4)
O5W—H51···O6'^iii^	0.84 (1)	2.07 (2)	2.823 (8)	148 (3)
O5W—H52···O2^iv^	0.85 (1)	1.94 (1)	2.792 (3)	175 (3)
O6W—H61···O6^i^	0.83 (1)	2.22 (3)	2.751 (8)	122 (3)
O6W—H62···O4W	0.84 (1)	1.88 (2)	2.642 (7)	150 (4)

Symmetry codes: (i) −*x*+2, −*y*+2, −*z*+1; (ii) *x*−1, *y*, *z*−1; (iii) −*x*+1, −*y*+2, −*z*+1; (iv) *x*−1, *y*, *z*.
